# The impact of continuous and intermittent ketogenic diets on cognitive behavior, motor function, and blood lipids in TgF344-AD rats

**DOI:** 10.18632/aging.205741

**Published:** 2024-04-12

**Authors:** Jennifer M. Rutkowsky, Zabrisky Roland, Anthony Valenzuela, An B. Nguyen, Heui Hye Park, Natalie Six, Ilknur Dursun, Kyoungmi Kim, Pamela J. Lein, Jon J. Ramsey

**Affiliations:** 1 Department of Molecular Biosciences, School of Veterinary Medicine, University of California, Davis, CA 95616, USA; 2 The MIND Institute, School of Medicine, University of California, Davis, CA 95817, USA; 3 Center for Neuroscience, University of California, Davis, CA 95616, USA; 4 Department of Physiology, School of Medicine, Istinye University, Istanbul 34396, Turkey; 5 Department of Public Health Sciences, School of Medicine, University of California, Davis, CA 95616, USA

**Keywords:** ketogenic diet, Alzheimer’s disease, cognitive behavior, motor function, lipids

## Abstract

Studies suggest that ketogenic diets (KD) may improve memory in mouse models of aging and Alzheimer’s disease (AD). This study determined whether a continuous or intermittent KD (IKD) enhanced cognitive behavior in the TgF344-AD rat model of AD. At 6 months-old, TgF344-AD and wild-type (WT) littermates were placed on a control (CD), KD, or IKD (morning CD and afternoon KD) provided as two meals per day for 2 or 6 months. Cognitive and motor behavior and circulating β-hydroxybutyrate (BHB), AD biomarkers and blood lipids were assessed. Animals on a KD diet had elevated circulating BHB, with IKD levels intermediate to CD and KD. TgF344-AD rats displayed impaired spatial learning memory in the Barnes maze at 8 and 12 months of age and impaired motor coordination at 12 months of age. Neither KD nor IKD improved performance compared to CD. At 12 months of age, TgF344-AD animals had elevated blood lipids. IKD reduced lipids to WT levels with KD further reducing cholesterol below WT levels. This study shows that at 8 or 12 months of age, KD or IKD intervention did not improve measures of cognitive or motor behavior in TgF344-AD rats; however, both IKD and KD positively impacted circulating lipids.

## INTRODUCTION

Alzheimer’s disease (AD) is the most common cause of dementia in the United States, affecting an estimated 12% of the population over the age of 65 [1]. The disease is characterized by the formation of amyloid beta (Aβ) plaques, neuronal tau tangles, neuroinflammation, and neuronal and synaptic loss [2]. Brain glucose hypometabolism and mitochondrial dysfunction precede these pathologies, leading to speculation that metabolic changes induced by a ketogenic diet may be an effective strategy for mitigating or slowing the progression of this disease [3, 4].

Ketogenic diets, which are depleted of carbohydrates, induce metabolic shifts similar to calorie restriction or fasting, resulting in increased fatty acid oxidation and elevated circulating ketone bodies, including β-hydroxybutyrate (BHB) [5]. Although glucose is the primary fuel source for the brain, ketone bodies can readily cross the blood-brain barrier and serve as a significant alternative energy source [6]. Further, BHB has been shown to act as a regulatory metabolite with potential relevance to aging through its modulation of lipolysis, oxidative stress, inflammation, and epigenetic modifications [7].

A few studies have evaluated the effect of a ketogenic diet on age and neurodegenerative disease-related cognitive decline. It has been shown that a continuous ketogenic diet (KD) initiated in middle age (12 months) can extend lifespan and improve both cognitive and motor behavior in 26-month-old mice [8]. Similarly, an intermittent ketogenic diet (KD) started at 12 months of age decreased mid-life mortality and improved cognitive behavior in aged mice [9]. Additionally, a 12-week KD intervention enhanced working memory in both young (4 months) and old (20 months) Fischer 344 x Brown Norway F1 Hybrid (FBNF1) rats [10]. A few studies have been completed with KD in mouse models of AD. A one-month KD in PSAPP mice improved motor performance without affecting amyloid beta load in the brain or muscle [11], while a four-month KD intervention in 5XFAD mice improved spatial and working memory coincident with reduced amyloid plaque deposition [12]. Another study found that consumption of a KD for 3 months in PSAPP and Tg4510 mice improved motor behavior but did not alter measures of memory [13]. While these studies suggest that a KD may be of some benefit in slowing AD progression in mouse models of AD, additional studies in other species are needed to determine the generalizability of these observations. In rats, there is some evidence that a KD may improve cognitive behavior. Measures of cognition were improved in 20-month-old FBNF1 rats consuming a KD for 12 weeks [10]. Consumption of a ketone ester for 5 days also improved a measure of cognition in Wistar rats [14]. However, feeding a KD for 8 weeks to Sprague Dawley rats infused with amyloid-β did not enhance measures of memory [15]. Thus, it remains to be determined whether long-term consumption of a ketogenic diet can mitigate declines in cognitive or motor behavior in a rat model of AD. Therefore, the current study aimed to determine whether a KD improves cognitive or motor behavior in the TgF344-AD rat. Aβ plaque deposition and tau-hyperphosphorylation increases in an age dependent manner in this model (TgF344-AD), which correlate with impaired spatial learning (15 and 24 months) and object memory (24 months) [16]. Another goal of the study was to determine whether an intermittent KD (IKD), intended to induce increases in blood ketones for part of each day as occurs with calorie restriction, is also capable of altering cognition or motor behavior. While a continuous KD is dietarily restrictive, an IKD may provide increased diet flexibility and improve compliance [17]. Blood lipids were also measured to determine the impact of sustained consumption of a KD or IKD on circulating triglycerides, free fatty acids, and cholesterol in this rat AD model. At 6 months of age, male and female TgF344-AD and wild-type littermate rats were placed on a two meal per day control diet (CD), KD or IKD (morning meal CD, afternoon meal KD). Comprehensive testing was performed to assess various aspects of cognitive and motor behavior at 8 and 12 months of age (at ages when cognitive function deficits are reported to emerge [18]); blood lipids and AD biomarkers were measured at 12 months of age.

## RESULTS

### Ketogenic diet and AD transgene expression altered body weight and KD diet interventions altered blood β-hydroxybutyrate levels

**Figure 1:**
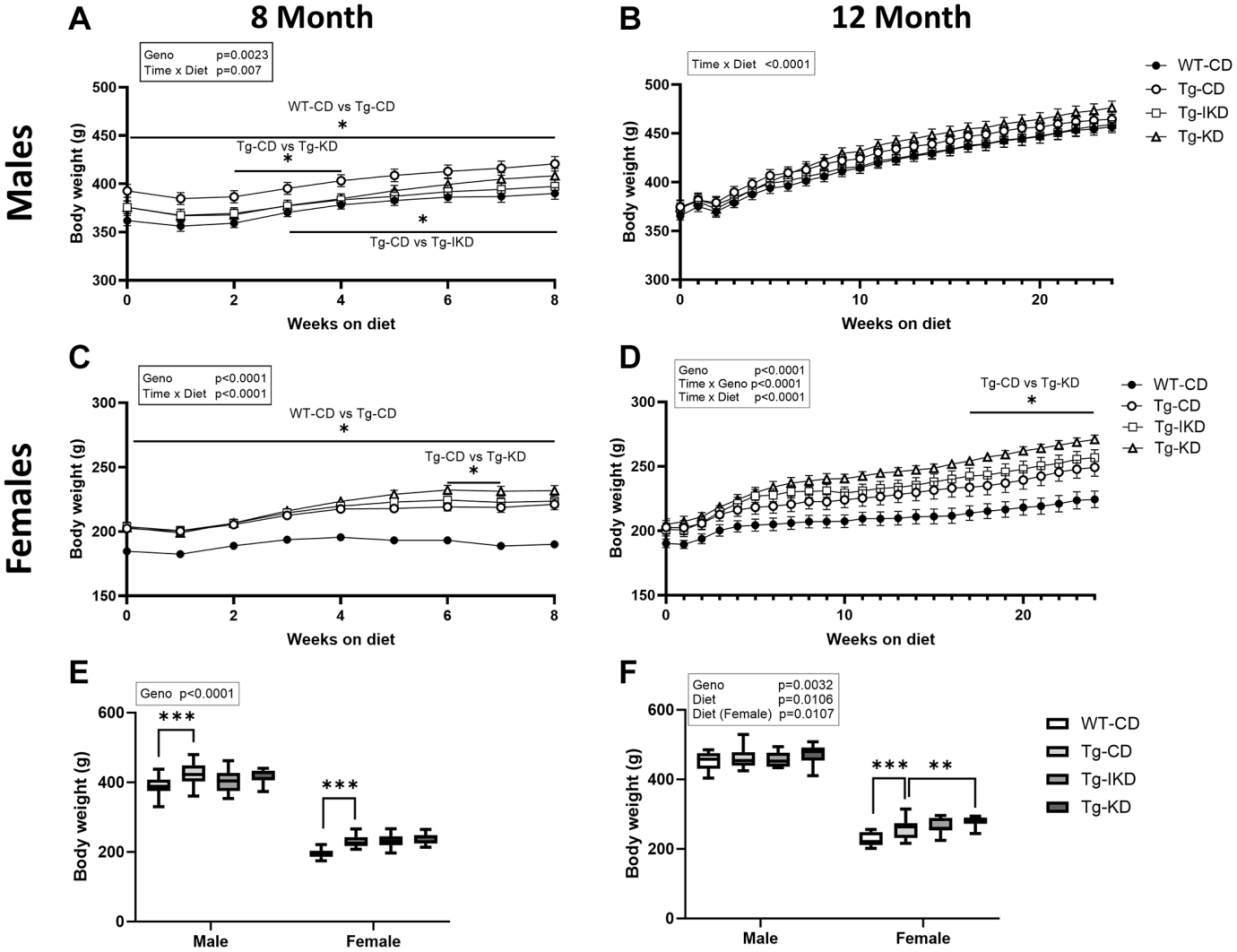
**Body weight in wild type (WT) and TgF344-AD (Tg) rats on control diet (CD), intermittent ketogenic diet (IKD) or ketogenic diet (KD).** (**A**–**D**) Mean body weights ± SEM of male (**A**, **B**) and female (**C**, **D**) rats fed CD, IKD, and KD fed rats throughout the 2 (8-month, **A** and **C**) and 6-month (12-month, **B** and **D**) diet intervention (*n* = 14–15/group). Both sexes and diet time courses showed significant time effect (*p* < 0.0001). Body weights at termination at 8 (**E**) or 12-months (**F**) of age demonstrated a significant sex (*p* < 0.0001) effect. ^∗^*p* < 0.05 and ^∗∗∗^*p* < 0.001 indicate significant differences between genotypes or diets based on repeated measures two-way ANOVA followed with Tukey’s post-hoc pairwise comparisons.

Body weight increased in both male and female rats over the course of the study regardless of genotype or dietary intervention (Figure 1A–1D). Comparing CD groups over time, genotype effects in body weight were observed as early as 6 months of age (at the start of diet intervention) in the 8-month male and female groups (Figure 1A, 1C), and there was a time x diet interaction (*p* < 0.01) between AD transgenes (Tg) diet groups in the 8- and 12-month male and female groups (Figure 1A–1D) suggesting the longitudinal changes of body weight over time were different between those diet groups. Furthermore, animals expressing AD transgenes (Tg) were significantly heavier at the end of the study than wild type (WT) littermates at both 8 and 12 months of age (Figure 1E, 1F; *p* < 0.0001 and *p* < 0.05 respectively). Specifically, female Tg rats consuming a CD weighed significantly more than WT littermates at 8 and 12 months of age, while Tg males on the CD only showed significant differences from WT at 8 months of age. At 12 months, female Tg animals weighed significantly more on KD compared to a CD, with body weights of female Tg animals on IKD intermediate to the other groups. In contrast, no significant differences were observed between diet groups in Tg male rats on the diets for 2 or 6 months.

**Figure 2:**
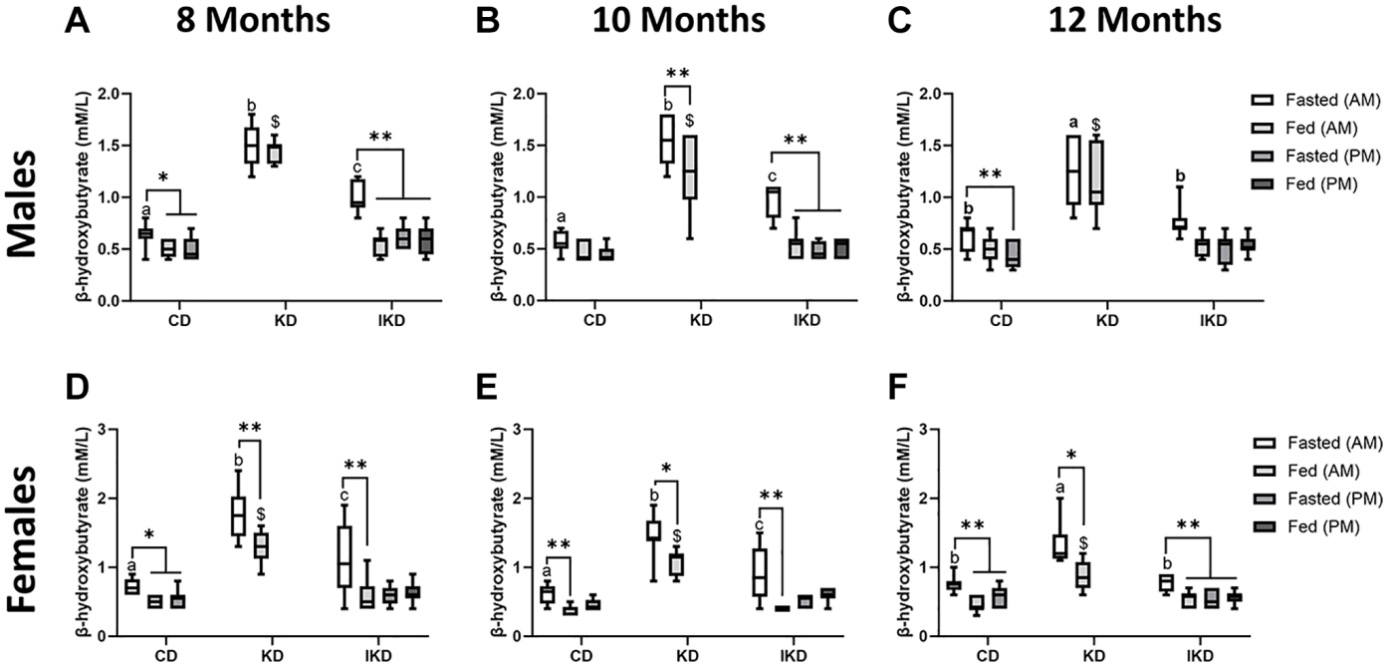
**Elevation of circulating level of β-hydroxybutyrate (BHB) with IKD and KD in TgF344-AD rats.** Mean circulating BHB levels (± SEM) were measured in male (**A**–**C**, *n* = 7–10/group) and female (**D**–**F**, *n* = 8/group) rats fed control diet (CD), intermittent ketogenic diet (IKD) or ketogenic diet (KD). Pre- and postprandial measurements were taken at 8 (**A**, **D**), 10 (**B**, **E**) and 12 (**C**, **F**) months of age (2, 4, or 6 months after initiation of diet). Data were analyzed by repeated measures two-way ANOVA followed with the Tukey’s post-hoc pairwise comparisons. ^*^p < 0.05 and ^**^p < 0.01 indicate significant differences between timepoints within a diet group. Different letters of a, b, or c denote difference (*p* < 0.05) between diets at the AM fast. ^$^ denote difference (*p* < 0.05) between diets at Fed AM timepoint. Both sexes showed significant overall diet and time effect at 2, 4 and 6 months (*p* < 0.0001).

Blood β-hydroxybutyrate measurements were taken from TgF344-AD animals of the 12-month Tg group at 8, 10, and 12 months of age. Ketone measurements were not taken in CD after the evening meal, nor in KD group pre- or post-evening meal, as the diet composition consumed in the evening matched that of the morning meal. Prior to the morning meal, blood ketone levels were highest in the KD-fed animals, lowest in the CD-fed animals, with IKD being intermediate. These levels were significantly different among all diet group in males and females at 8- and 10-months (Figure 2A, 2B, 2D, 2E). However, at 12 months, the IKD ketone levels were no longer different from the CD and only the KD group was different from the other diet groups (Figure 2C, 2F). Postprandially, ketone levels in both the CD and IKD groups dropped in response to the morning CD meal, but the KD-fed group remained significantly elevated in the males and only showed a postprandial decrease in the females. IKD fed animals did not show an increase in blood ketone levels postprandially to the evening KD meal (Figure 2., Fed PM) at any age.

### KD and IKD ameliorated elevated plasma lipids in Tg-F344 AD rats

Plasma triglyceride, free fatty acid (FFA), total cholesterol, and HDL and non-HDL (LDL and VLDL) cholesterol levels were measured in blood samples at 12 months of age (Table 1). Females were found to have significantly lower lipid levels compared to males (sex main effect, *p* < 0.05). A genotype main effect for all lipids except triglyceride and non-HDL cholesterol was observed, along with a diet main effect involving triglycerides and cholesterol (total, HDL, and non-HDL, *p* < 0.001). Male Tg animals were found to have significantly higher triglyceride (*p* < 0.05) compared to WT, while female Tg rats had higher total cholesterol, FFA, and HDL compared to WT (*p* < 0.01). IKD and KD reduced triglyceride compared to Tg-CD and cholesterol levels (total, HDL, and non-HDL) compared to both the Tg and WT CD males. In females, the KD decreased cholesterol levels (total, HDL, and non-HDL) compared to both the Tg and WT CD groups and the IKD decreased total and HDL-cholesterol compared to the Tg CD rats.

**Table 1:** Influence of Alzheimer’s transgene and diet on plasma lipids.

	**Male**				**Female**				***p*-value**				
	**WT-CD**	**Tg-CD**	**Tg-IKD**	**Tg-KD**	**WT-CD**	**Tg-CD**	**Tg-IKD**	**Tg-KD**	**Sex**	**Geno**	**Diet**	**S × G^1^**	**S × D^2^**
Triglyceride (mg/dl)	183.3 ± 15.7^b^	257.4 ± 22.1^a^	177.4 ± 14.3^b^	134.7 ± 9.9^bc^	150.2 ± 22.2^bc^	149.1 ± 16.7^bc^	135.4 ± 9.7^bc^	100.8 ± 5.8^c^	<0.001	ns	<0.001	ns	ns
Free-Fatty Acid (mEq/l)	0.42 ± 0.05^ab^	0.56 ± 0.04^a^	0.51 ± 0.04^a^	0.51 ± 0.01^a^	0.28 ± 0.04^b^	0.5 ± 0.04^a^	0.46 ± 0.03^a^	0.43 ± 0.03^ab^	0.015	0.001	ns	ns	ns
Total Cholesterol (mg/dl)	179.7 ± 7.2^ab^	199.1 ± 9.9^a^	117.3 ± 3.2^cd^	104.1 ± 3.1^d^	121.9 ± 4^c^	158.2 ± 6.1^b^	113.1 ± 3.3^cd^	95.7 ± 2.5^d^	<0.001	0.011	<0.001	ns	0.012
HDL-C (mg/dl)	138.7 ± 6.8^ab^	154.4 ± 8.3^a^	85.8 ± 3^cd^	78.1 ± 3.5^d^	100.4 ± 3.3^c^	125 ± 5.1^b^	86.8 ± 4^cd^	77.4 ± 2.2^d^	<0.001	0.014	<0.001	ns	0.019
non HDL-C (mg/dl)	41 ± 2^ab^	44.7 ± 2.5^a^	31.6 ± 1.6^c^	25.9 ± 1.1^cd^	28.8 ± 1.8^c^	33.3 ± 1.7^bc^	26.3 ± 2.7^cd^	18.3 ± 0.7^d^	<0.001	ns	<0.001	ns	ns

Values with different superscript letters statistically differ between groups (*p* < 0.05). Abbreviation: ns: not significant; S x G^1^: Sex x Genotype; S x D^2^: Sex x Diet.

### KD reduced a subset of plasma AD biomarkers in female, but not male TgF344-AD rats

**Figure 3:**
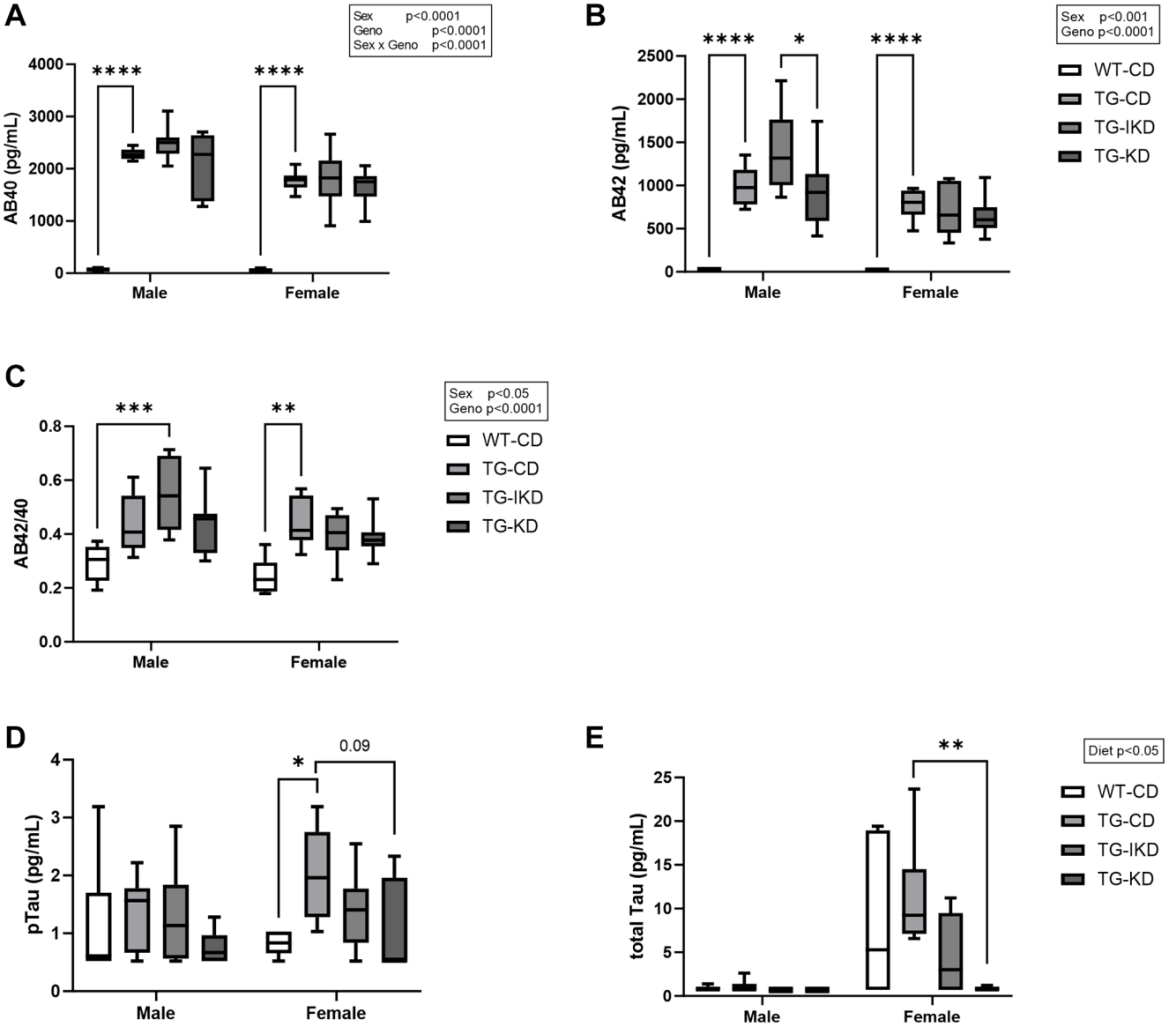
**Plasma level of amyloid beta (Aβ) and phosphorylated Tau 181 (pTau).** (**A**) Mean circulating Aβ40, (**B**) Aβ42, (**C**) Aβ42/42 ratio, (**D**) pTau, and (**E**) total Tau were measured in male and female rats fed control diet (CD), intermittent ketogenic diet (IKD) or ketogenic diet (KD) (*n* = 6–8/group). Aβ40, Aβ42, and Aβ42/40 ratio data were analyzed by repeated measures two-way ANOVA with Tukey’s post-hoc pairwise comparisons. Phosphorylated and total Tau data were analyzed using Kruskal-Wallis’s test with Dunn’s post-hoc to compare differences between diet groups within sex. ^*^p < 0.05, ^**^p < 0.01, ^***^p < 0.001, ^****^p < 0.0001 indicate significant differences between groups.

Amyloid beta (Aβ) 40, Aβ42, phosphorylated Tau 181 (pTau) and total Tau (tTau) were measured in plasma samples collected at 12 months of age (Figure 3). Males were found to have significantly elevated Aβ40, Aβ42 and Aβ42/40 ratio compared to females (Figure 3A–3C, sex main effect, *p* < 0.05). While there was a genotype main effect with Tg-CD animals exhibiting significantly elevated Aβ40, 42 and Aβ2/40 ratio compared to WT-CD, neither IKD nor KD ameliorated this difference. In females, pTau was significantly increased in Tg fed a CD (*p* < 0.05) compared to WT-CD and there was a trend (*p* = 0.09) for KD to mitigate elevated pTau levels (Figure 3D). In addition, KD significantly reduced tTau compared to Tg-CD female rats (Figure 3E, *p* < 0.01).

### KD or IKD did not improve motor coordination impairment in TgF344-AD rats

**Figure 4:**
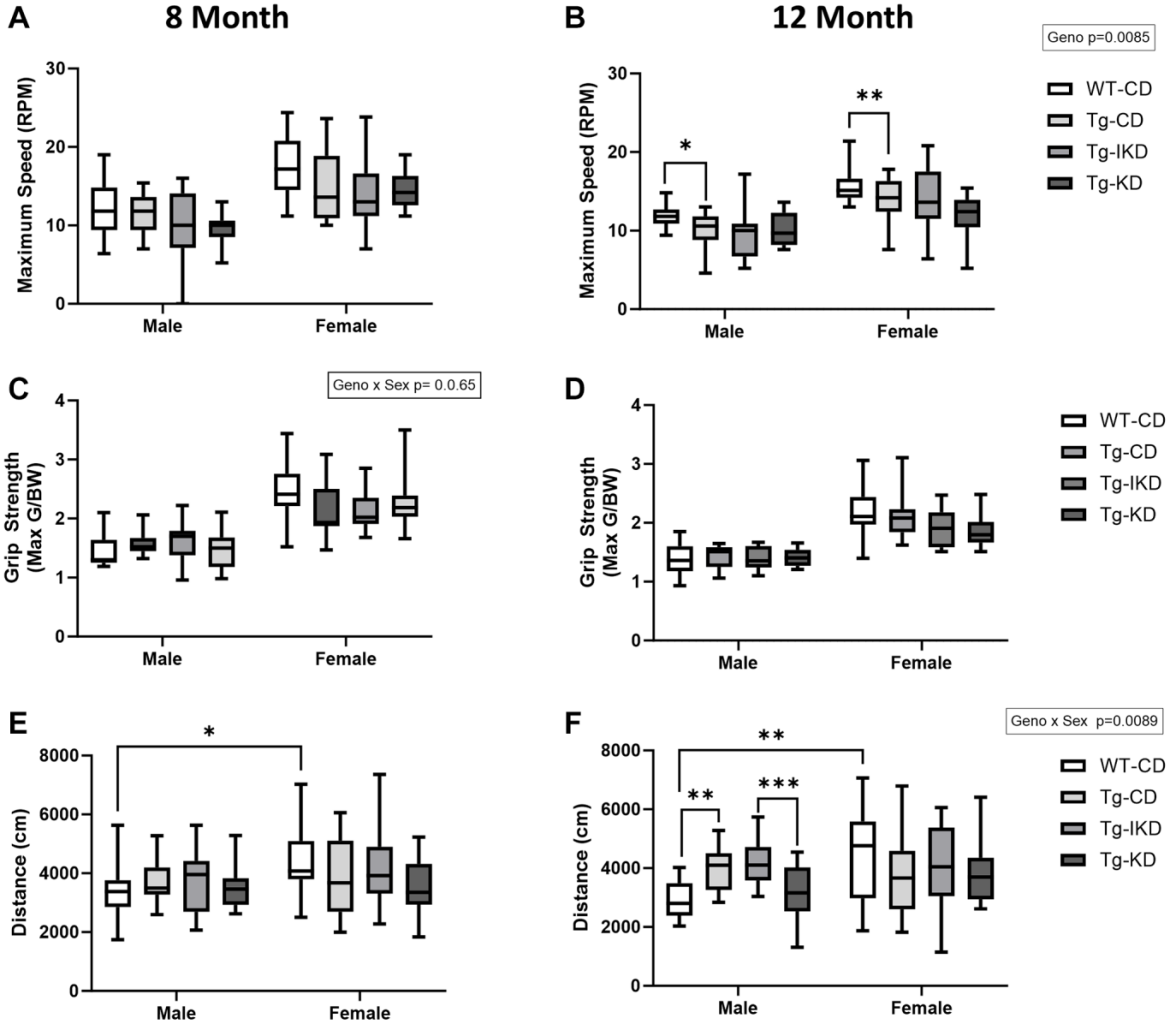
**Motor coordination and muscle strength in TgF344-AD rats fed IKD and KD diet.** Rotarod maximum speed (**A**, **B**, *n* = 9–14/group), grip strength (**C**, **D**, *n* = 13–15/group), and total distance traveled in open field (**E**, **F**, *n* = 13–15/group) were assessed in wild type (WT) and Tg433-AD (Tg) rats. Rats were assessed at 8 (**A**, **C**, and **E**) or 12 (**B**, **D**, and **F**) months of age and data were analyzed by two-way ANOVA followed with the Tukey’s post-hoc pairwise comparisons. ^*^p < 0.05, ^**^p < 0.01, ^***^p < 0.001, ^****^p < 0.0001. Diets: control (CD), intermittent ketogenic (IKD) and ketogenic (KD). Both timepoints (8 and 12 month) showed a significant sex effect in rotarod, grip strength test, and open field (*p* < 0.05).

Motor behavior and muscle strength were assessed using the rotarod and grip strength tests (Figure 4). Female rats performed better on the rotarod test than males at both 8 and 12 months of age, as indicated by a longer time to fall (data not shown, *p* < 0.0001) and increased maximum speed (Figure 4A, 4B, *p* < 0.0001). At 12 months of age, Tg animals had an overall lower maximum speed (Figure 4B, *p* < 0.01) when compared to WT-CD animals. Specifically, maximum speed was significantly lower in both male and female Tg CD versus WT CD rats (Figure 4B). As body weight differed between genotypes and diet groups and may impact rotarod performance, time to fall and maximum speed were corrected by body weight but significant differences remained the same as prior to correction (data not shown). Finally, rotarod performance did not differ between Tg diet groups at either 8 or 12 months of age.

Grip strength performance was not impacted overall by diet or genotype at either age tested. However, there was a sex effect where females exhibited improved grip strength (max G/BW) over males (Figure 4C, 4D, *p* < 0.001). Furthermore, there was a sex x genotype interaction at 8 months of age where female WT-CD tended to perform better than Tg-CD, but this was not observed in males.

In the open field test, females explored and covered more distance than males at both ages tested (Figure 4E, 4F, *p* < 0.05). At 12 months of age, there was a sex x genotype interaction where Tg males, but not females, traveled a greater distance than WT (*p* < 0.01). Further, Tg males on a KD diet covered significantly less distance than rats on an IKD (*p* < 0.001). There were no other differences among the Tg diet groups in the open field test at either 8 or 12 months of age.

### TgF344-AD animals demonstrated impaired spatial learning memory that was not mitigated by diet

**Figure 5:**
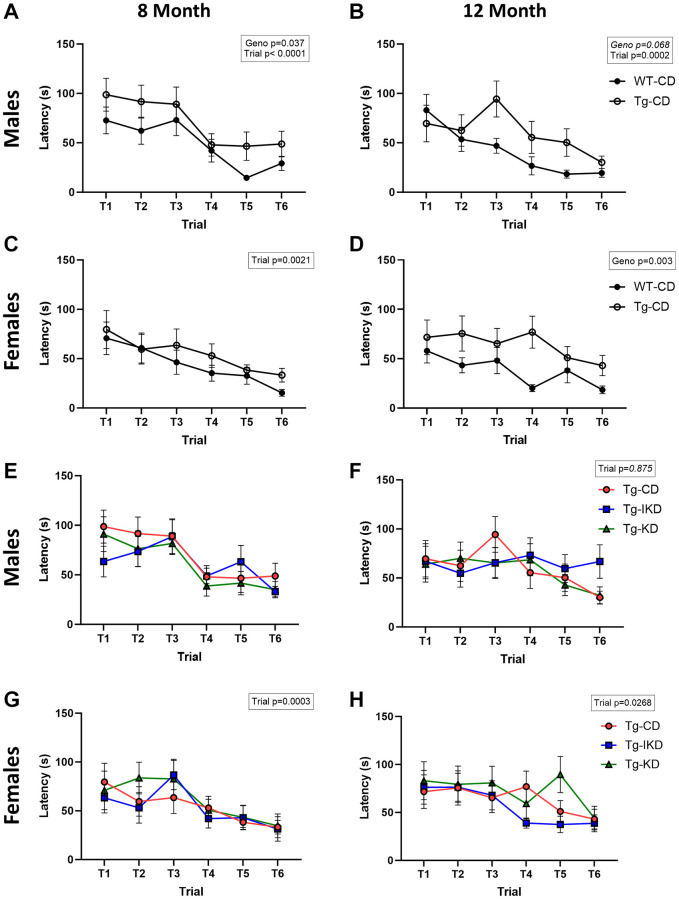
**Spatial learning in WT and TgF344-AD rats.** Female and male wild type (WT) and Tg433-AD (Tg) rats on a control diet (CD) were trained in the Barnes maze test at 8 or 12 (**A**–**D,*** n* = 14–15/group) months of age and latency to escape over trials reported. Spatial learning (latency to escape over trials) was determined in TgF344-AD rats fed a control (CD), intermittent ketogenic (IKD) and ketogenic (KD) at 8 or 12 (**E**–**H,*** n* = 14–15/group) months of age.

**Figure 6:**
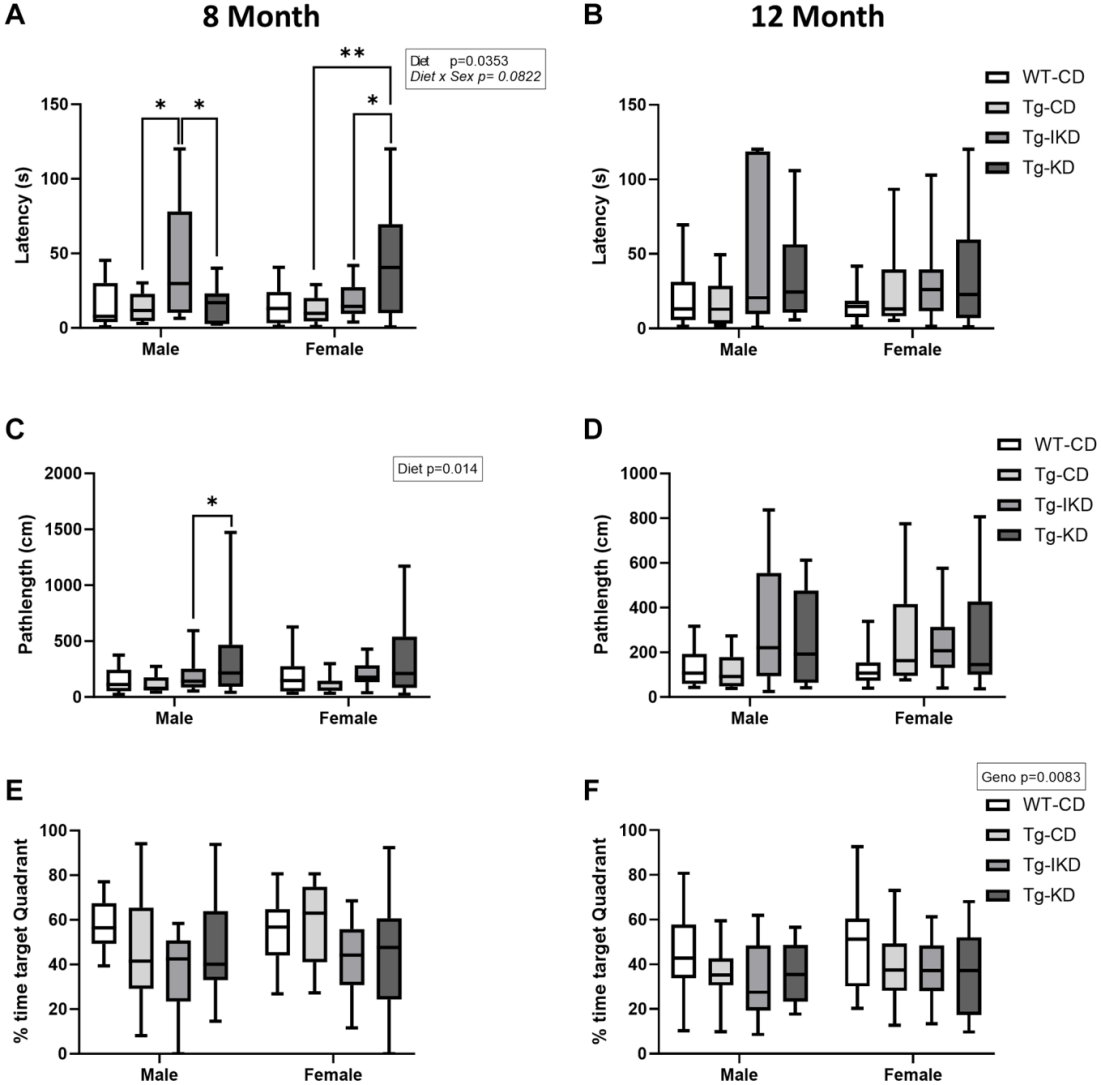
**Spatial memory in IKD and KD fed TgF344-AD rats.** Barnes maze latency to the target hole (**A**, **B**), pathlength to target hole (**C**, **D**) and time spent in the target quadrant (**E**, **F**) in the probe trial of the Barnes maze test was determined in wild type (WT) and Tg433-AD (Tg) rats. Behaviors were assessed at 8 (**A**, **C** and **E**, *n* = 11–15/group) or 12 (**B**, **D**, and **F**, *n* = 13–15/group) months of age and data were analyzed by two-way ANOVA followed with the Tukey’s post-hoc pairwise comparisons. ^*^p < 0.05, ^**^p < 0.01. Diets: control (CD), intermittent ketogenic (IKD) and ketogenic (KD).

At 8 months of age, Tg males on a CD showed spatial learning memory impairment during the training phase as assessed by the Barnes maze (Figure 5A, *p* < 0.05), while 12-month-old males showed a strong trend toward impaired spatial learning memory (Figure 5B, *p* = 0.068) compared to WT. In females, impaired performance in the Barnes maze test was observed in the 12-month-old, but not 8-month-old, rats (Figure 5C, 5D, *p* < 0.01). Neither KD nor IKD improved performance in the Barnes maze task and there were no differences between the CD, IKD, and KD groups in Tg male and female rats for Barnes maze training (Figure 5E–5H). During the probe trial, there was an overall genotype effect in which Tg rats at 12, but not 8, months of age spent significantly decreased % time in the target quadrant (Figure 6E, 6F, *p* < 0.01). There were significant (*p* < 0.05) diet effects on latency and pathlength to the target at 8 months of age in the Tg animals, and there was a trend for diet x sex interaction for latency (Figure 6A, 6C). However, these differences in the probe trial did not continue at 12 months of age and no differences between Tg diet groups were observed in the 12-month-old rats (Figure 6B, 6D).

### Measures of anxiety were not altered by the KD or IKD compared to CD in TgF344-AD rats

**Figure 7:**
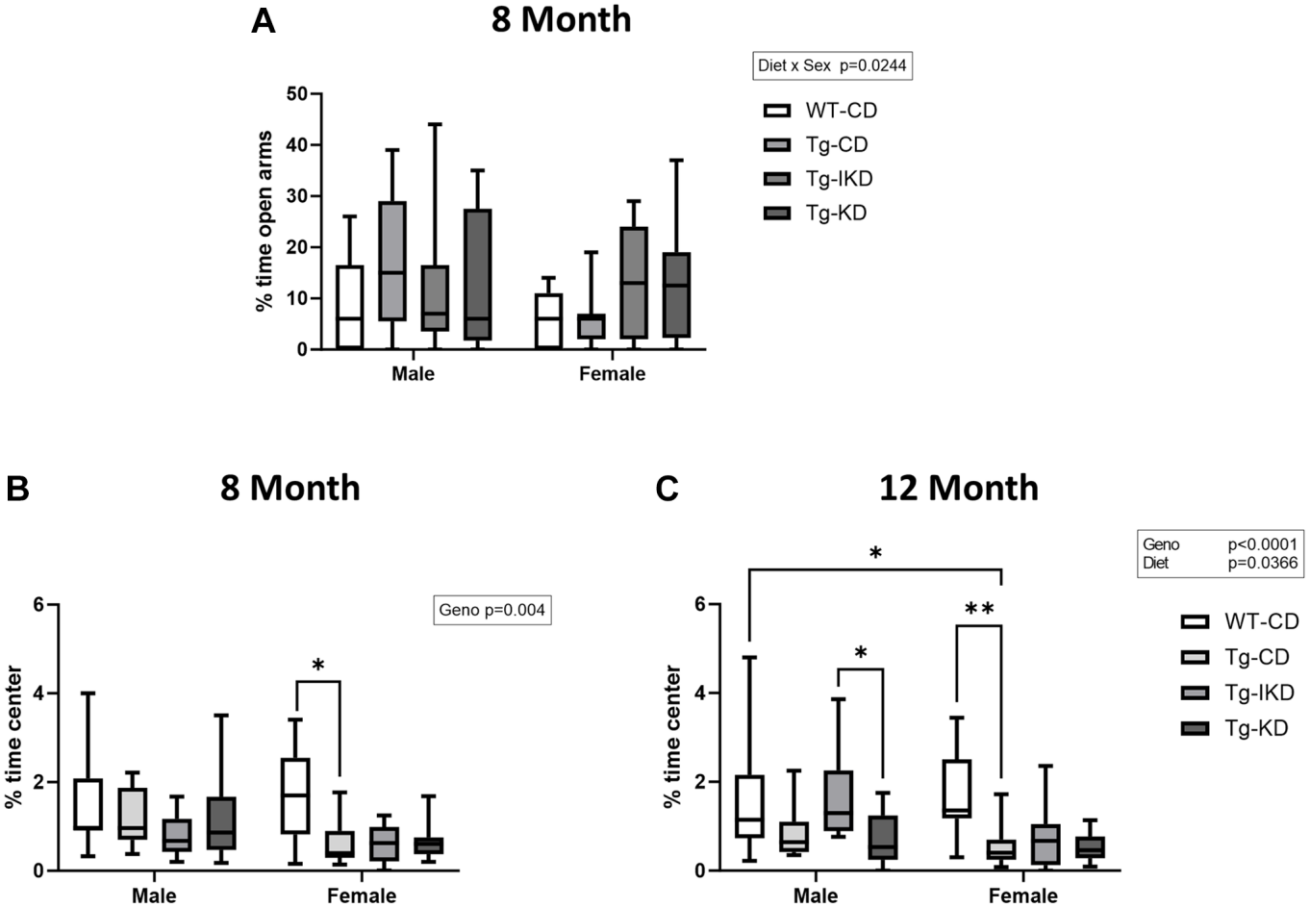
**Measure of anxiety in TgF344-AD (Tg) and wild type (WT) rats.** Performance in the elevated plus maze (**A**, *n* = 9–15/group) was assessed at 8 months of age and % time spent in the center of the open field was assessed at 8 (**B**, *n* = 9–12/group) and 12 (**C**, *n* = 10–14/group) months of age. Data were analyzed by two-way ANOVA followed with the Tukey’s post-hoc pairwise comparisons. Elevated plus maze (8 months of age) and % time spent in the center (8 and 12 months of age) demonstrated a significant sex (*p* < 0.05) effect. ^*^p < 0.05, ^**^p < 0.01, ^***^p < 0.001. Diets: control (CD), intermittent ketogenic (IKD) and ketogenic (KD).

To assess anxiety, 8-month-old rats were tested on the elevated plus maze (Figure 7A). In addition, time spent in the center of the open field was assessed as a potential indicator of anxiety in both 8- and 12-month-old rats (Figure 7B, 7C). There was no genotype or diet effect in the elevated plus test, although there was an overall effect of sex on % time spent in open arms with female rats tending to spend reduced time in the open arms compared to males (Figure 7A, *p* < 0.01). When using % time in the center of the open field as a measure of anxiety, Tg animals showed decreased time in the center compared to WT at both 8 and 12-months of age (Figure 7B, 7C, *p* < 0.05). In addition, there was a diet effect at 12 months of age (*p* < 0.05) with male Tg animals on a IKD diet spending significantly more time in the center than KD and a strong trend toward more time than CD (*p* = 0.0525).

## DISCUSSION

It has been demonstrated that a KD can improve both cognitive and motor behavior in rodent models of aging [8–10, 19]. There have also been reports that a KD can improve cognitive [12] and motor function [11, 13] in mouse models of AD. However, there has also been a study reporting no improvement in cognitive function with consumption of a KD in two mouse models of AD [4]. Intermittent fasting, which can trigger cycles of ketosis in laboratory rodents, has also been reported to improve cognition in a mouse model of AD [20], supporting the idea that intermittent periods of ketosis may also provide some benefit for mitigating changes in behavior with AD. This study aimed to evaluate whether a KD or IKD could improve cognition or protect motor function in the TgF344-AD rat model of Alzheimer’s disease. Our findings indicate that while both KD and IKD elevated circulating β-hydroxybutyrate (BHB) levels, neither diet improved muscle strength or motor coordination as determined by grip strength and rotarod performance. Additionally, neither intervention improved spatial learning memory in the TgF344-AD rats at 8 or 12 months of age. However, both diets decreased circulating lipid levels, which were elevated in the TgF344-AD model.

Epidemiological studies have highlighted the complex relationship between body weight and dementia, indicating that individuals who are overweight or obese have a higher risk of cognitive decline. However, it has also been observed that a decrease in body weight or being underweight can also precede or occur with the onset of dementia [21]. In this study, TgF344-AD rats were found to be heavier than their WT littermates despite being fed isocalorically matched meals. The Tg female animals had higher body weights than WT females at the termination of the study at both 8- and 12-months of age, while the Tg males had greater body weights than WT males at the 8-month of age study termination. These results are consistent with other studies that have reported increased body weights in female TgF344-AD compared to WT rats [22, 23]. In contrast, increased body weight in the male TgF344-AD rats was transitory since no differences in weight between genotypes were observed at 12 months of age and this finding is consistent with other studies that found no difference in body weight between TGF344-AD and WT male animals [23, 24]. The present study was unique, however, in that the rats were fed isocaloric amounts of diet. Thus, the increased body weight in the TgF344-AD rats was not due to increased food intake. Future studies are needed to measure energy expenditure and physical activity in these animals to determine whether altered energy expenditure is a factor contributing to weight gain, and potentially pathology, in the TgF344-AD female rats.

In this study we found plasma levels of Aβ40, Aβ42, and the Aβ42/40 ratio to be elevated in all TgF344-AD animals fed a CD compared to sex- and diet-matched WT animals at 12 months of age. In addition, plasma levels of pTau were increased in female TgF344-AD relative to female WT. These results are consistent with studies indicating that plasma levels of these AD biomarkers are positively correlated with amyloidosis and tau pathology in the brain and to cognitive impairment [25–27]. Similar to a recent study in human subjects with mild cognitive impairment [28], we found that the KD significantly decreased plasma levels of total Tau with a trend to reduce plasma pTau levels. Neither IKD or KD reduced plasma levels of Aβ40 or Aβ42 or the Aβ42/40 ratio. Additional studies evaluating the Aβ and pTau levels in the brain may be more sensitive indicators of KD effects on AD pathologies in the TgF344-AD rat model.

As expected, the continuous KD significantly elevated both fed and fasting ketone measurements, while intermediate measurements were observed in the IKD group [15, 29]. It was also observed that TgF344-AD rats exhibited elevated blood lipids compared to age- and sex-matched WT littermates. Prior research has indicated that elevated levels of triglycerides, glucose, total cholesterol, and LDL are associated with increased AD risk [30, 31]. Additionally, a correlation between elevated cholesterol levels and AD pathology has been observed [32]. Lipids and their associated lipoproteins, particularly apolipoproteins, have been shown to associate with Aβ in human plasma and may serve as a carrier for Aβ [33]. Further, individuals with the E4 variant of apolipoprotein E (APOE-E4) are predisposed to high cholesterol levels and late onset familial or sporadic forms of AD [34]. Whether elevated cholesterol levels in the Tg-344-AD rat precede Aβ pathology and are an added risk factor or if they are a result of APP and presenilin overexpression remains to be determined. Previous research has shown that a KD did not significantly alter circulating triglycerides or total cholesterol in Wistar rats fed ad lib [35] and meal fed aged C57BL/6 mice [8]. However, in this study, it was found that the KD, and to a lesser extent the IKD, reduced TgF344-AD cholesterol (total, HDL, and non-HDL) levels in males and females and triglycerides in males when compared to WT. This may be in part due to an upregulation of primary bile synthesis and bile secretion pathways that shuttle cholesterol to bile acids, which are then excreted [36]. Further, there are indications that KD may lower triglyceride levels in normal weight men [37] and obese or overweight patients with type 2 diabetes [38]. Additional work is needed to determine whether the KD-related decreases in blood lipids contribute to improved health in the TgF344-AD rats and it remains to be determined whether a KD will produce similar changes in blood lipids in other animal models of AD, and potentially humans.

While loss of muscle strength and coordination are not typically associated with AD until later stages, there are some indications that loss of motor function correlate with cognitive decline in AD [39]. Therefore, the effect of the ketogenic diet (KD) on motor coordination and muscle strength was examined using rotarod and grip strength. The 8-month AD animals showed no impairment of motor coordination on the rotarod test (max speed), but there was an impairment by 12 months of age when compared to WT littermates. This is akin to what is seen with humans, with more robust changes in motor coordination at later stages of AD [40]. However, no deficit in muscle strength as assessed by the grip strength was observed in AD rats at 8 or 12 months of age, although there was a significant age-related decline. While higher grip strength is associated with better cognitive performance in humans [41], these animals may have been assessed too early in the disease progression to detect an impairment. KD has been reported to improve performance on the rotarod test in mouse models of AD [11, 13]. However, in this study, TgF344-AD rats on KD or IKD performed equally well to their CD fed counterparts. It should also be noted that observed deficits in motor function between the AD and WT mice consuming the control diet were relatively small in magnitude and assessing motor function at an older age (>15 months) may be needed to determine if diet mitigates more severe muscle coordination and muscle strength deficits.

Previous studies have demonstrated that a KD can improve object and working memory in aging mice and rats [8, 9, 19]. Furthermore, KD was shown to improve spatial learning memory and working memory in one study with a mouse model of AD [12], but the KD did not improve measures of memory in another study using a different mouse model of AD [13]. The current study aimed to determine if KD or IKD positively impacted cognition in the TgF344-AD rat. Similar to what was shown by Bac et al. [18], the TgF344-AD rats had a spatial memory deficit at 8 months of age in males, and this deficit was present in both males and females by 12 months of age. While there was no difference between the WT and Tg at 8 months of age during the probe trial, there was a significant decreased % time in the target quadrant at 12 months. These results indicate that the Tg spatial learning impairment is subtle and may best be elucidated with the repeated measure of the training phase. The results of the present study are similar to what was observed by Cohen et al., who reported an increase in the number of errors during training and in the probe trial [16]. The more robust cognitive impairment in TgF344-AD animals in Cohen et al. may be due to their group rotating the maze 90° each day to change the position of the escape box, making the test more difficult and adding another layer of learning. While a diet effect was seen at 8 months of age (latency and pathlength), these effects were in the direction of impaired spatial learning and memory with KD and these significant changes with diet did not persist at 12 months of age. The results of the present study indicate that the KD and IKD did not improve spatial memory/learning at ages when impairments began to emerge in the TgF344-AD rats. However, further studies would be needed to determine if the KD influences cognitive behavior at later life when deficits in the TgF344-AD rats may become more severe.

One limitation with the present study is that behavior testing in this AD model (TgF344-AD) is challenging due to reduced movement/activity (Novel Object Recognition and Y maze tests, data not shown) and balance issues observed in elevated plus (more pronounced in males and potentially related to heavier weight), which limited the cognitive and anxiety tests that could be completed. In addition, improved cognitive and motor behavior in Tg344-AD rats by IKD or KD may be challenging to detect as the impairments are modest and detection may be complicated by the reduced mobility and balance observed in the 8- and 12-month-old TgF344-AD rats. Further, due to the sample size (*n* = 9–15 per group), we did not perform Bonferroni correction for multiple testing for the motor function and cognitive tests and a future study with a larger sample size to allow for these corrections may be warranted. Another limitation of the study is that feeding the diet as 2 meals per day resulted in a period of fasting, especially between the evening and morning meal, which led to a significant increase in the morning pre-meal blood ketone levels in the CD group compared to postprandial measurements. It has previously been reported that feeding mice once per day an amount of diet equivalent to *ad libitum* intake induces meal eating and a daily period of fasting [42]. Our study indicates that feeding rats an *ad libitum* amount of diet split into two daily feedings also induces meal feeding in many animals that produces an overnight fast sufficient to induce ketosis with the CD. This elevation in ketone levels, while short term, may have impacted cognitive measures to diminish differences between WT-CD and Tg-CD fed animals, making it more difficult to detect diet induced differences.

The present study demonstrated that TgF344-AD rats had impaired motor coordination and spatial learning memory deficits, which were exacerbated at the latter time point. Moreover, the IKD or KD did not improve motor coordination or spatial learning memory compared to the CD. However, KD, and to a lesser extent IKD, mitigated elevations in plasma lipids in the TgF344-AD rats. Furthermore, the KD diet decreased plasma levels of total Tau in females. Future studies evaluating the effectiveness of KD in older Tg rats, as well as evaluation of the effect of KD on brain Aβ, Tau, and neuro-inflammatory markers, are warranted.

## METHODS

### Animals

Male TgF344-AD rats, hemizygous for two human transgenes, APPswe and PSEN1DE9 [16], were obtained from Dr. Robert Cohen (Emory University) to establish a breeding colony at UC Davis. Rats were housed in polycarbonate cages on racks in a HEPA filtered room maintained on a 12-hour light-dark cycle where temperature (22–24°C) and humidity (40–60%) were controlled. Health checks were performed daily. To produce litters of transgenic (Tg) and wild-type (WT) animals with genotypes evenly distributed between the sexes, hemizygous male TgF344-AD rats were bred with female WT Fischer (CDF) rats obtained from Charles River Laboratories.

Harem breeding trios of two females and one male were used to generate the required animals. Between postnatal day 2–4, pups were tattooed with coded identification numbers using nontoxic animal tattoo ink (Ketchum Manufacturing Inc, Luzerne, NY, USA). At 7–10 days of age, tail tips were clipped for genotyping as described below. At 21 days of age, animals were weaned, separated by sex, and housed 3–4 per cage. At ~2 months of age animals were co-housed (2/cage) until 6 months of age. Some animals were single housed during this period due to odd cohort/sex animal number. Enrichment in the form of a plastic house was provided for animals showing signs of stress (i.e., excess porphyrin production). If the genotype of an adult needed to be reassessed, a small ear punch was collected. All animal protocols were approved by the UC Davis Institutional Animal Care and Use Committee.

### Genotyping

DNA was extracted from tail tips or ear punches prior to amplification using PCR. The PCR amplification generated two products: one from the transgene (if present) and one from the rat genome. The rat genome product served as a control to ensure the integrity of the extracted DNA. The PCR reaction was performed with three primers: one antisense primer matching a sequence within PrP that is present in both the MoPrP vector and the genome (5′: GTG GAT ACC CCC TCC CCC AGC CTA GAC C), one sense primer specific to the transgene cDNA (APP: 5′: CCG AGA TCT CTG AAG TGA AGA TGG ATG), and a second sense primer specific to the genomic PrP coding region that had been removed from the MoPrP vector (5′: CCT CTT TGT GAC TAT GTG GAC TGA TGT CGG). PCR amplification was carried out in a reaction volume of 25 μL, consisting of 8.3 μL nuclease-free water, 12.5 μL GoTaq Green master mix, 0.4 μL of each primer, and 3 μL of extracted DNA. The thermal cycler was programmed to include an initial denaturation step of 3 minutes at 94°C, followed by 30 cycles of denaturation at 94°C for 1 minute, annealing at 55°C for 1 minute, extension at 72°C for 2 minutes, and a single final extension of 5 minutes at 72°C. Electrophoresis was performed on the PCR products using a 1.5% (w/v) agarose gel in 1 × TAE buffer, running at 150 V for approximately 30 minutes. For visualization, SyberSafe was added to the gel mix and a 100 BP DNA ladder was loaded as a marker. The gels were imaged on a GelDoc system using a SyberSafe compatible tray, and the PrP and transgene bands were located at 400 and 750 base pairs (bp), respectively.

### Diet

Before the study began, rats were co-housed when possible and provided unrestricted access to standard rat chow (Teklad 2019; Envigo, Livermore, CA, USA). At 6 months of age, rats were individually housed, balanced by body weight, and randomly assigned to one of three groups: control (CD), intermittent (IKD), or ketogenic (KD). Each group was provided with two meals per day (~8AM and 5PM) with a daily calorie allowance divided equally. Rats in the IKD group received a morning meal of CD and an evening meal of KD. Throughout the study, males and females were given 63 kcal/day and 42 kcal/day, respectively. While food intake (food remaining at the end of each meal) was not measured, it was noted that all males and most females completed each meal before being provided the next. The control diet consisted of 10% protein, 74% carbohydrate, and 16% fat. Control diet was pelleted by adding 5% water w/w and processing using Colorado mill equipment MILL-3 Pellet Mill. The ketogenic diet consisted of 10% protein, less than 0.5% carbohydrate, and 89.5% fat (Table 2). Diets were prepared in-house at least every 4 months and stored at 4°C.

**Table 2:** Diet composition.

**Ingredients (g/kg)**	**Control**	**Ketogenic**
Protein, of which	112.5	193.7
Casein	111	191
DL-methionine	1.5	2.7
Carbohydrates, of which	722	−
Corn starch	490	−
Maltodextrin	132	−
Sucrose	100	−
Fat, of which	70	652
Soybean oil	70	70
Lard	−	582
Mineral mix^a^	35	24.1
Vitamin mix^b^	10	17.6
Cellulose	48	85
TBHQ	0.014	0.13
K+ phosphate monobasic	2.4	−
Ca+ phosphate dibasic	−	19.3
Ca+ carbonate	−	8.2

^a^The CTL diet used the Teklad TD94046 mineral mix and the KD used the Teklad TD98057 mineral mix. ^b^All diets used Teklad TD40060 vitamin mix.

### Ketone measurements

Blood β-hydroxybutyrate levels were measured using a Precision Xtra glucose and ketone monitoring system (Abbott, Chicago, IL). Blood was obtained via a tail nick in both the fasted (immediately prior to AM or PM meal) or fed (3 hours post-prandial) animals. Measurements were taken 2, 4 and 6 months after initiation of diet from TgF344-AD animals in the 12-month-old cohorts.

### Plasma lipid analysis

Following behavior tests, animals were terminated under 5% isoflurane anesthesia with medical O_2_/compressed air (25/75%) at 2 LPM (Vaporizer Sales and Service, Rockmart, GA, USA). Blood was collected from cardiac puncture in 10 ml EDTA vacutainer tubes (Becton Dickinson, Franklin Lakes, NJ, USA). Plasma was isolated by centrifuging blood at 2000 × G for 15 minutes at 8°C and sent to the UC Davis Nutrition Department for analysis. Enzymatic colorimetric assays were completed using kits according to the manufacturer’s instructions: free fatty acids (FujiFilm Wako Diagnostics Mountain View, CA, USA), triglycerides and total cholesterol (Fisher Diagnostics, Middletown, VA, USA). LDL and VLDL were precipitated using reagents from Abcam (Cambridge, MA, USA), and supernatant HDL-C was measured (Fisher Diagnostics, Middletown, VA, USA). Markers of Alzheimer’s disease pathology were measured using a Human Amyloid Beta and Tau 4-Plex Custom Assay (NF-HNABTMAG-68K, MilliporeSigma, Burlington, MA, USA) on a Luminex^™^ 200 system (Luminex, Austin, TX, USA) by Eve Technologies Corp. (Calgary, Alberta, Canada).

### Comprehensive motor and cognitive testing

To evaluate the effects of dietary interventions on motor and cognitive behavior, a battery of tests was used, including elevated plus maze (day 1), open field (day 2), grip strength (day 6), rotarod (day 7–10), and Barnes maze (day 13–16). Two time-points were assessed: 8-months-old (2 months on diet) and 12-months-old (6 months on diet) in separate cohorts of animals. Prior to testing, all equipment was cleaned with a 10% bleach solution followed by 70% ethanol, and 70% ethanol was used to clean between testing trials. When moving the animals to a different room for testing, they were given 30 minutes to acclimate to their new environment to minimize stress. A white noise machine was used to mask any extraneous noise in the room.

### Elevated plus maze

Elevated plus maze was conducted to assess anxiety levels in rats as previously described [43]. For this test, a black opaque plastic elevated plus maze with open (50 × 10 cm) and closed arms (50 × 10 × 30 cm) with a square center (10 × 10 cm) elevated to ~70 cm, was used. Each rat was placed in the center of the maze, facing an open arm, and allowed to freely roam for 5 minutes. EthoVision XT15 (Noldus, Wageningen, the Netherlands) was used to track the amount of time the rat spent in each arm of the maze. Lower anxiety levels are indicated by an increased % time in the open arms of the elevated plus maze. 12-month-old animals were not assessed due to the high fall rate during testing.

### Open field

Open field was employed to evaluate gross locomotor activity and exploration patterns in a blue plastic box (55 × 55 × 55 centimeters). Following Berg et al. [44] the animals were placed in the center of the arena and allowed to explore for 30 minutes, and their movement patterns, including total distance traveled and % time spent in the center (28 × 28 cm), were recorded and analyzed automatically using EthoVision XT15 (Noldus, Wageningen, the Netherlands). Increased total distance traveled is a positive indicator of improved voluntary locomotor activity while a decrease in the amount of time in the center is a measure of anxiety.

### Grip strength

To assess grip strength (modified from [45]), rats were positioned to grasp the wire grid attached to an Imada (DST-11 Northbrook, IL, USA) push-pull scale with their forelimbs. Once the forepaws grasped the grid, the rat was pulled horizontally away from the meter until its grasp was broken. Each animal performed two sets of three trials, with a maximum of 30 seconds per trial. A rest period of at least 10 minutes was given between sets, and the highest G-force (G) value from each trial was recorded and the data reported as G force divided by body weight in grams (G/BW). An increase G/BW is indicative of increased grip strength.

### Rotarod

To test motor coordination, an accelerating rotarod protocol modified from [46] was conducted using a Rotamax-4 lane (7 cm) Rota-Rod (Columbus Instruments, Columbus OH, USA). Clean towels were placed at the bottom of each lane to cushion falls from the rod. Prior to testing, rats were given 3 days of training where they were given 10 minutes to walk on the rotating rod at a constant speed of 4.0 revolutions per minute (rpm). Any rat that fell more than 5 times during the last training session was excluded. On the fourth day, rats were placed on the rod, with an initial speed of 4 rpm and accelerated by 1 rpm every 6 seconds to a maximum of 40 rpm. The test day consisted of 3 test sessions per rat (intertrial interval of ~30 minutes) with the latency to fall and max speed recorded. An increase in latency to fall and max speed are indicators of improved motor coordination.

### Barnes maze

Barnes maze testing was performed to evaluate spatial learning and memory and modified from Koulousakis et al. [47]. During the first three days, each rat underwent two training trials per day with an intertrial interval of ~15 minutes, followed by a probe trial on day four. The maze consisted of a black circular platform (92 cm diameter) which was elevated ~80 cm from the ground, with 20 equally spaced 5 cm holes around the perimeter and a black escape box under one of the holes. Visual cues were placed on panels located approximately 25 cm away from the maze and an overhead LED light source was used to illuminate the maze (~700 lux).

On the first day, an inverted polycarbonate mouse cage was used to guide each rat to the hole with the escape box prior to the first trial. For each of the training trials, the rat was placed in the center of the arena with the lights off and covered with a black bucket. After 10 seconds, the lights were turned on, and the bucket was removed. Each rat was allowed to explore for 3 minutes or until it entered the escape box. If the rat failed to enter the escape box within three minutes, a polycarbonate mouse cage was used to guide it into the escape box. When a rat entered the escape box, the lights were turned off and it was allowed to rest for 30–45 seconds before being returned to its cage.

On the fourth day (probe day), the escape box was removed, and the rats were allowed to explore for 2 minutes. These sessions were recorded in EthoVision XT 15 (Noldus, Wageningen, the Netherlands), and the latency to target, pathlength to target, and % time spent in target quadrant were measured. A decreased latency to target and pathlength to target as well as an increased % time in target quadrant are indicators of improved spatial learning and memory.

### Statistical analysis

All statistical analyses were performed using GraphPad Prism 10 (GraphPad Software Inc., San Diego, CA, USA). Descriptive statistics are expressed as mean ± standard error of mean (SEM) for normally distributed variables and median ± interquartile range (IQR) for pTau and total Tau not normally distributed. Outliers within each group were identified using the ROUT method, a robust nonlinear regression-based method [48], and removed from statistical analysis. Prior to statistical inference tests, the analysis of residuals was performed to validate the normality and homoscedasticity assumptions using QQ and residual plots as well as D’Agostino and Pearson test for normality. Where appropriate, log or square root transformation was applied to data to approximately conform to normality and homogeneity of variances prior to statistical inference tests. Missing values of plasma pTau and total Tau below the limit of detection (LOD) were imputed as one-half (1/2) of the marker-specific minimum of the detectable values prior to statistical analysis. Body weight and Barnes maze training data were analyzed using repeated measures ANOVA, followed by Tukey’s post-hoc test for pairwise group comparisons to assess differences between genotypes (WT-CD and Tg-CD) or diets (Tg-CD, Tg-KD, and Tg-IKD) and time. For cross-sectional analyses of all other data, two-way ANOVA was used to evaluate differences between genotypes or diets within sex and age (8 or 12 months) and its genotype-diet interaction, followed by Tukey’s post-hoc test for pairwise group comparisons. One-way ANOVA followed by the Tukey’s or Kruskal-Wallis’s test with Dunn’s post-hoc (if data were not normally distributed) was used to compare differences between diet groups within a sex as appropriate. For plasma lipid analysis we used the Bonferroni correction to adjust for multiple comparisons. Statistical significance was determined at two-sided *p* < 0.05.
